# Veterinary Medical Association of New Jersey

**Published:** 1893-01

**Authors:** W. H. Cooper

**Affiliations:** Secretary


*Veterinary Medical Association of New Jersey.* The Veterinary Medical Association of New Jersey held its December meeting at 11:30 Thursday morning, December 8th, at the City Hotel, New Brunswick. President James C. Dustan, of Morristown, presided, and Secretary W. H. Cooper, of Trenton, kept the minutes. About fifteen members were present when the roll was called. This number was afterwards increased to twenty. The minutes of the last regular meeting were read and approved. Secretary Cooper read his report, in which he made a number of recommendations, with a view of getting the Association out of its present rut. The report was ordered to come up for further discussion. The Secretary was instructed to forward application blanks to certain veterinary surgeons whose names had been suggested.

Dr. Cooper, one of the delegates to the United States Association at Boston, reported attending the meeting and having had a most excellent time. Numerous instructive papers were read, and the delegates were handsomely entertained. There was a feeling that more delegates should have attended from local societies. Mr. F. B. Kilmer, chemist at Johnson & Johnson's, was introduced, and invited the members of the Association to visit the establishment of Johnson & Johnson and view the process of manufacturing surgical appliances. The invitation was accepted.

The topic of "Tuberculosis" was discussed to some extent. Dr. W. B. E. Miller, of Camden, who is in the employ of the Agricultural Department at Washington, said that in the first year of his inspection in Philadelphia he had found that about $2\frac{1}{2}$ or 3 per cent. of all the animals inspected had tuberculosis. Since then the percentage had fallen about one per cent., not because there was less tuberculosis, but because the farmers and stock raisers recognize it now and do not send animals affected to the market. But there is scarcely a day that tuberculosis is not found. There is more tuberculosis in South Jersey to the square inch than in any other part of the country he had visited. It exists to a great extent throughout the whole of New Jersey.

Dr. Hunt, of the State Board of Health, thought the disease was abating. It is only abating because the farmers do not send animals so affected to the markets. Dr. Miller instanced the case of diseased animals seized in Camden a few days ago. There were nine, four of which had tuberculosis, one had actinomycosis, and the other four were free from disease. These cattle had been picked up by the dealer about the country. He also told of visiting a slaughter house in Camden and finding several cases, one of them being the handsomest case of tuberculosis he ever saw. Fifteen cattle were in the herd and all were killed. Five of them were suffering from tuberculosis. He believed that from what he had seen that tuberculosis existed to as great an extent now as ever, and it is now high time for the veterinarians of the State to appeal to the Legislature for the passage of a law that would prevent absolutely the placing of the flesh of such cattle and others that have been exposed to tuberculosis upon the markets.

Dr. Julius Gerth thought that this subject should be under the charge of the State Board of Agriculture instead of the State Board of Health.

Dr. J. W. Hawk, of Newark, spoke upon the subject, and said there was no question that the disease was raging throughout the State. He knew of one herd on Orange Mountain, the milk from which was being sent to market. In reference to Dr. Gerth's idea, he said the question was how to make the members of the Legislature appreciate the dangers of the situation, and it was doubtful which body would have the most power.

Dr. Miller said at the outset the work was in charge of the Board of Agriculture. The work was done effectively, although somewhat expensively, but he believed the cost was one of the best investments the State ever made. He thought the work was best done by the Board of Agriculture. He thought tuberculosis should be classed with pleuro-pneumonia and stamped out in the same way. Dr. Hunt, of the State Board of Health, has declared that there is no power to confiscate cattle suffering with tuberculosis, because it was not fully decided whether or not tuberculosis is or is not contagious. He believed, and was upheld by leading pathologists, that it was communicable to human beings. Dr. Hawk said the question was, which Board could get the largest appropriation from the State. It is certain that a diseased animal cannot give wholesome milk. The State Board of Health does not go far enough; it quarantines the cattle, but does not order their destruction. Dr. Gerth said Dr. Hunt was a stickler for the technicalities of the law. He said the agriculturists are directly interested in preserving the health of the cattle, the Board of Health only indirectly. Therefore the Board of Agriculture should have the matter in charge.

President Dustan agreed with the remarks already made and thought a committee should be appointed to draft resolutions expressing the opinion of this Association as to the medical action in the matter. Tuberculosis is on the increase in his district.

Dr. Miller invited the members to attend the Sanitary Association at Lakewood to-morrow and Saturday, and said the question of " Tuberculosis " would be discussed on Saturday.

Dr. Gerth said it had been proved that calves from healthy mothers would develop tuberculosis if fed with milk from tuberculous cows.

On motion a vote was taken whether or not the Society should take action on the subject. It was decided that it should.

Dr. Gerth moved that the President appoint a committee of three to draft resolutions on the subject of Tuberculosis to be submitted at this meeting for consideration. Dr. Higgins, Dr. Eisenhardt and Dr. Hurley were appointed as such Committee.

Letters of regret at being unable to be present were read from Dr. Rush S. Huidekoper of New York and Dr. W. Horace Hoskins of Philadelphia.

The Treasurer's report showed receipts to date of $198.19; expenditures $115.81, balance on hand $82.38.

The report of the Secretary was taken up and the recommendations discussed. Dr. Miller opposed the recommendations for a monthly meeting of the Board of Trustees but favored the recommendation that a committee on registration be appointed to see that none but regular veterinarians are registered and permitted to practice. He declared that he knew that men were registered and practicing illegally and it seemed to be nobody's business to look after it.

The Secretary's report recommending the appointment of Committees or Registration, Legislation and Judiciary was adopted. At two o'clock the Society adjourned for dinner and afterward visited the factory of Johnson & Johnson's, where the different processes of manufacturing surgical appliances were explained by Mr. Kilmer. The visit proved very interesting to the members of the Association.

The President upon reassembling appointed the following Committees :

*Legislation*, Dr. Miller, Dr. Hawk, Dr. Higgins, Dr. Cooper, Judge Kilburn.

*Registration*, Dr. Gerth, Dr. Axford, Dr. Brown.

*Judiciary*, Dr. Hawk, Dr. Wittpenn, Dr. Elliott.

*Essayists for next meeting*, Dr. Gerth, Dr. Elliott, Dr. Eisenhart.

The following were proposed for membership : Dr. William T. I. McLaughlin, Jersey City; Dr. C. E. Anderson, Elizabeth; Dr. J. P. Matthews, Princeton.

The Tuberculosis Committee presented the following, which was read by Dr. Higgins :

*Whereas*, the disease known as Bovine Tuberculosis, is transmittable to man through the consumption of meat and milk ; and is known also to be increasing among our dairy cattle, causing a serious financial loss to the stock growers and agriculturists of this State, therefore

*Be it Resolved* ; that it is the sense of this meeting that proper legislation authorizing the State authorities to co-operate with the United States Bureau of Animal Industries, in eradicating contagious animal diseases is absolutely necessary, especially as to tuberculosis and that as a matter of policy, the supervision of this work should be transferred to the State Board of Agriculture, said body being directly interested.

The report was unanimously adopted.

President Dustan made a short address congratulating the society upon its growth and the good it has already accomplished. He reviewed the rise of the veterinary profession and claimed that in the scientific treatment of disease and in pathology the veterinary profession now stood at the head.

The next meeting of the association was appointed to be held at Trenton.

The following members were present:

W. B. E. Miller, Camden; J. W. Hawk, Newark; J. C. Dustan, Morristown A. Brown, Windsor; W. H. Cooper, Jersey City; L. P. Hurley, Hopewell; J. C. Higgins, New Brunswick; B. F. King, Little Silver; H. W. Rowland, Newark; C. T. Fithran, Camden; J. Gerth, Newark; J. M. Everett, Hackettstown; M. M. Stage, Dover; A. W. Axford, Naughright; H. W. Elliott, Newark; J. N. Wittpenn, Jersey City; A. C. Eisenhart, Asbury; S. Lockwood, Woodbridge.

W. H. Cooper, *Secretary.*

